# Delivered dose quantification in prostate radiotherapy using online 3D cine imaging and treatment log files on a combined 1.5T magnetic resonance imaging and linear accelerator system

**DOI:** 10.1016/j.phro.2020.06.005

**Published:** 2020-07-13

**Authors:** Charis Kontaxis, Daan M. de Muinck Keizer, Linda G.W. Kerkmeijer, Thomas Willigenburg, Mariska D. den Hartogh, Jochem R.N. van der Voort van Zyp, Eline N. de Groot-van Breugel, Jochem Hes, Bas W. Raaymakers, Jan J.W. Lagendijk, Hans C.J. de Boer

**Affiliations:** University Medical Center Utrecht, Department of Radiotherapy, 3508 GA, Utrecht, The Netherlands

**Keywords:** Dose accumulation pipeline, Prostate cancer, Intrafraction cine-MR, Soft-tissue motion tracking, MR-linac, Treatment log files

## Abstract

**Background and purpose:**

Monitoring the intrafraction motion and its impact on the planned dose distribution is of crucial importance in radiotherapy. In this work we quantify the delivered dose for the first prostate patients treated on a combined 1.5T Magnetic Resonance Imaging (MRI) and linear accelerator system in our clinic based on online 3D cine-MR and treatment log files.

**Materials and methods:**

A prostate intrafraction motion trace was obtained with a soft-tissue based rigid registration method with six degrees of freedom from 3D cine-MR dynamics with a temporal resolution of 8.5–16.9 s. For each fraction, all dynamics were also registered to the daily MR image used during the online treatment planning, enabling the mapping to this reference point. Moreover, each fraction’s treatment log file was used to extract the timestamped machine parameters during delivery and assign it to the appropriate dynamic volume. These partial plans to dynamic volume combinations were calculated and summed to yield the delivered fraction dose. The planned and delivered dose distributions were compared among all patients for a total of 100 fractions.

**Results:**

The clinical target volume underwent on average a decrease of 2.2% ± 2.9% in terms of D99% coverage while bladder V62Gy was increased by 1.6% ± 2.3% and rectum V62Gy decreased by 0.2% ± 2.2%.

**Conclusions:**

The first MR-linac dose reconstruction results based on prostate tracking from intrafraction 3D cine-MR and treatment log files are presented. Such a pipeline is essential for online adaptation especially as we progress to MRI-guided extremely hypofractionated treatments.

## Highlights

• First delivered dose reconstruction based on six degrees of freedom motion from online 3D cine-MR imaging and treatment log files.
• For most fractions no significant target dose decrease observed.
• Few fractions with outlier motion due to bladder/rectal filling causing large dose deviations.
• Automated fast dose reconstruction crucial for future MRI-guided treatments.

## 1 Introduction

With the introduction of methods to quantify intrafraction prostate motion during radiotherapy [1], [2], [3], [4], its impact on dose blurring has become more conspicuous [5]. While the impact of prostate intrafraction motion over a fully fractionated scheme was found to be relatively small [6], current routine clinical practice and subject of recent studies is geared towards hypofractionated treatments [7], [8] as well as the use of reduced margins [9]. This has led to increasing interest of the radiotherapy community for accurate reconstruction of the delivered dose to the prostate and surrounding Organs At Risk (OAR).

While several methods for dose reconstruction are described in literature, popular approaches are based on combining the simulated or delivered beams with the prostate intrafraction motion, obtained by tracking implanted fiducial markers with kilovoltage (kV) imaging [10], megavoltage (MV) imaging [5] or using magnetic implanted markers with the Calypso system [6]. Although these methods can provide a fair estimation of the delivered dose to the prostate, they lack soft tissue intrafraction information of surrounding OAR [2] and can therefore not be used to accurately reconstruct the delivered dose in the OARs.

With the introduction of combined Magnetic Resonance Imaging (MRI) and linear accelerator systems new possibilities have arrived, which include the ability to obtain MRI during radiotherapy delivery with a greatly improved soft tissue contrast over X-ray based imaging [11]. This improved soft tissue contrast is especially advantageous in the abdomen, as cone beam computed tomography (CBCT) is hindered by organ motion artefacts and low tissue contrast [12]. By using the MR-images obtained during the beam-on period, soft tissue information of the target location and surrounding OAR can be extracted from these images, allowing for accurate tracking of the prostate and surrounding OAR. Eventually, real-time plan adaptation will be enabled and based on this information [13]. In addition, real-time plan adaptation may lead to the use of reduced planning target volume (PTV) margins, while possibly capturing large prostate intrafraction motion outliers [14].

Previously we have published our method to calculate the impact of prostate intrafraction motion on the planned dose distribution as obtained from 3D cine-MR, but used cine-MR images that were acquired after radiotherapy, essentially simulating an online MRI-guided treatment [15]. The impact on delivered dose in MRI-guided prostate patients based on anterior-posterior (AP) and cranial-caudal (CC) translations extracted from intrafraction 2D cine-MR imaging and treatment log files was recently presented [16].

In this study we apply our dose accumulation pipeline on the first prostate patients treated on a 1.5T MR-linac in our clinic. It is the first study that combines a validated soft-tissue contrast based tracking algorithm with 3D cine-MR to extract six degrees of freedom (translational and rotational) motion in conjunction with the treatment log files. These files contain the timestamped information of the linac parameters during delivery and are required to perform accurate reconstruction of the delivered dose during each fraction.

## 2 Material and methods

Five low-intermediate prostate patients were registered as part of an institutional review board approved registration and imaging study and underwent prostate stereotactic body radiotherapy with 20 daily fractions of 3.1 Gy. The patients were treated on a 1.5T MR-Linac (Elekta Unity) with simultaneous 3D cine-MR imaging during the beam-on period of the treatment. These cine-MR images were acquired using a 3D balanced turbo field echo (bTFE) sequence. A total of 88 cine-MR imaging data sets were acquired with a temporal resolution of 16.9 s and spatial resolution of 0.9 × 0.9 × 2.0 mm, while after additional improvements to the cine-MR sequence (such as removing fat suppression) the remaining 12 fractions over two patients were acquired with a temporal resolution of 8.5 s and spatial resolution of 0.8 × 0.8 × 2.2 mm. Technical details of the sequence are provided in Tables S1 and S2 of the supplementary material.

### 2.1 Treatment planning

Prior to the first fraction the patients underwent CT and MR simulation scans. Plans were created in the Elekta Monaco v5.40.01 treatment planning system using a 5 beam setup. The minimum number of monitor units per segment was 5 MU with an average of 51.6 segments per plan. A calculation grid spacing of 3 mm was used with a statistical dose uncertainty per segment of 3%. The Elekta Unity has a multi-leaf collimator (MLC) with a leaf width of 7 mm. A total of 160 leafs are present which travel in the cranio-caudal direction and 7 MV flattening filter free beam energy is used.

The clinical target volume (CTV) for these five patients contained the body of the prostate. The PTV structure prescribed at 57 Gy was created using an isotropic margin of 5 mm around the CTV. Moreover, an extended boost volume (EBV) prescribed at 62 Gy was formed by using a margin of 5 mm margin in the caudal, left - right (LR) and anterior directions while excluding the rectum and bladder in an attempt to ensure proper coverage of the CTV while sparing the adjacent OARs. The clinical constraints are presented in Table 1.

**Table 1:** Prostate planning constraints, where EBV is the extended boost volume and PTV as the planning target volume.

VOI	Constraints		
EBV_62	V58.9 Gy	>	99%
PTV_57	V54.15 Gy	>	99%
Rectum	V62Gy	<	1 cm^3^
	V60Gy	⩽	5%
	V40Gy	⩽	50%
Bladder	V62Gy	<	1 cm^3^
	V60Gy	⩽	10%
	V40Gy	⩽	50%
Femur heads	V40Gy	⩽	50%

### 2.2 Online workflow

After positioning the patient on the treatment couch, a daily pre-treatment MR scan (PRE) was obtained. Plan adaptation using full replanning was then performed based on the patient’s anatomy on the PRE. Planning was performed on a pseudo-CT based on the PRE generated by bulk-density assignment of the patient body and bony anatomy. Following an automatic contour propagation, a clinician manually adjusted the prostate and OAR contours for the adapted plan and after plan calculation, a position verification (PV) scan was acquired. If the CTV was not covered by the planning margins the plan was rigidly shifted and dose was recalculated using Monaco’s build-in option “optimize weights” to match the new prostate position. Treatment delivery was then started along with simultaneous 3D Cine-MR imaging, acquiring a whole 3D volume (dynamic) every 16.9 or 8.5 s dependent on the scan protocol used. Directly after radiotherapy delivery a post-treatment scan (post) was acquired. The PRE, PV, and POST scans were all acquired with a T2-weighted 3D sequence with a duration of 2 min. Technical details of these sequences are provided in Table S3 in the supplementary material.

### 2.3 Registration

Intrafraction motion of the prostate was determined by using a rigid registration algorithm based on soft tissue contrast with six degrees of freedom (i.e. 3D translations along the LR, AP and CC directions and respective rotations about these axes). This method uses the daily CTV delineation of the prostate body on the first cine-MR dynamic to determine the region of interest. Subsequent dynamics were then rigidly registered to the first dynamic based on soft tissue contrast of the prostate, yielding one rigid transformation per timepoint. The methodology of the registration algorithm has previously been described and validated [17]. This registration method was also used to rigidly register the first cine-MR dynamic to the daily PRE scan, transferring the local cine-MR motion to the reference coordinate system and thus yielding the prostate intrafraction motion over the entire period that the patient was positioned on the treatment table.

### 2.4 Dose reconstruction

The intrafraction dose accumulation was performed by combining the motion information obtained from the intrafraction 3D cine-MR dynamics with the linac machine log files. These log files store all parameters during delivery and each log file contains the relevant machine parameters—including MLC/gantry positions and Monitor Units—required to reconstruct each given treatment fraction at a 25 Hz frequency.

Each log file was split based on the time interval of the 3D dynamics (16.9 s or 8.5 s), yielding several partial plan/3D volume combinations. For each partial plan, a pseudo-CT volume was created by bulk density assignment of the corresponding cine-MR. This was performed with the same method used during the initial planning. The body contour and bony structures were kept constant within each fraction to avoid unrealistic motion of the anatomy, as the motion registration results accurately describe only the high dose region around the prostate. The partial dose was calculated using the research version of the Elekta GPUMCD dose engine [18, ] using identical settings to the clinical Monaco system. Then, for the purpose of dose accumulation, the partial dose was warped back to the reference PRE volume by using the inverse rigid transformation. Finally, for each fraction the partial doses were summed leading to the accumulated fraction dose (INTRA) which was compared to the respective daily planned (REF) dose. For comparison purposes, the fraction INTRA doses were also scaled by the number of fractions to the total treatment dose level (20 × 3.1 Gy). The comparison was performed by extracting statistics of various clinical Dose Volume Histogram (DVH) points for the targets and OARs between REF and INTRA.

For all calculations workstations with Intel Xeon CPUs, at least 32 GB RAM and Nvidia GTX Titan cards were used.

## 3 Results

The average preparation time during each fraction prior to radiation delivery was 26 ± 5.4 min, while radiation delivery took on average 5.5 ± 0.7 min per fraction. For five out of 100 fractions it was necessary to adjust the plan based on the intrafraction motion observed on the PV scan, for which the registration and dose accumulation reference was appropriately modified. Four of these cases were due to intrafraction motion and one case was due to technical difficulties.

### 3.1 Pipeline timings

The 3D registration algorithm took approximately 10.7 ± 2.5 s for each image pair to match the CTV region of interest and yield the underlying translations/rotations. The dose reconstruction took approximately 12 ± 0.6 s per dynamic volume/partial plan combination to calculate the dose and warp it to reference space.

### 3.2 Motion statistics

**Fig. 1:**
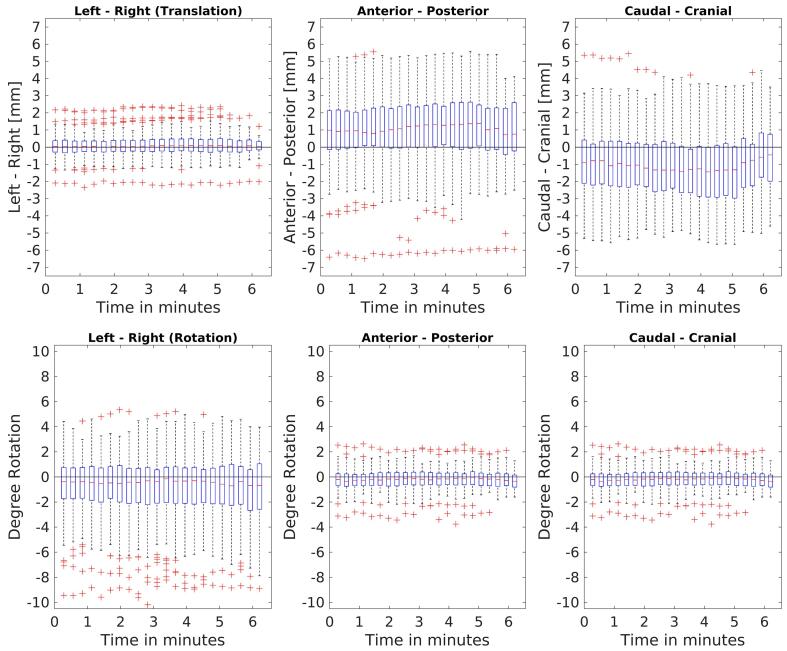
Boxplots of the intrafraction motion during beam-on period based on cine-MR with respect to the planned anatomy of the PRE scan. The boxplots at every timepoint contain 100 datapoints each, one per fraction. The boxplots above 4 min include gradually less datapoints equal to the fractions in which delivery was still active. The horizontal black lines indicate y = 0.

The bulk motion occurred within the approximately 30 min prior to radiation delivery, after which the prostate position seems to settle, with occasional motion events occurring during delivery on an individual basis. The mean ± SD translations (mm) and rotations (degrees) prior to radiation delivery were on average 0.1 ± 0.6 (LR), 0.9 ±1.9 (AP), −0.9 ± 2.0 (CC) and −0.7 ± 2.3 (LR), −0.2 ± 0.8 (AP), 0.0 ± 1.2 (CC) while during beam-on were 0.0 ± 0.2 (LR), 0.2 ± 0.9 (AP), −0.3 ± 1.0 (CC) and −0.1 ± 1.2 (LR), 0.1 ± 0.5 (AP), 0.1 ± 0.6 (CC) respectively. The motion used during each time point for dose accumulation is given in Fig. 1.

### 3.3 Dose accumulation

**Fig. 2:**
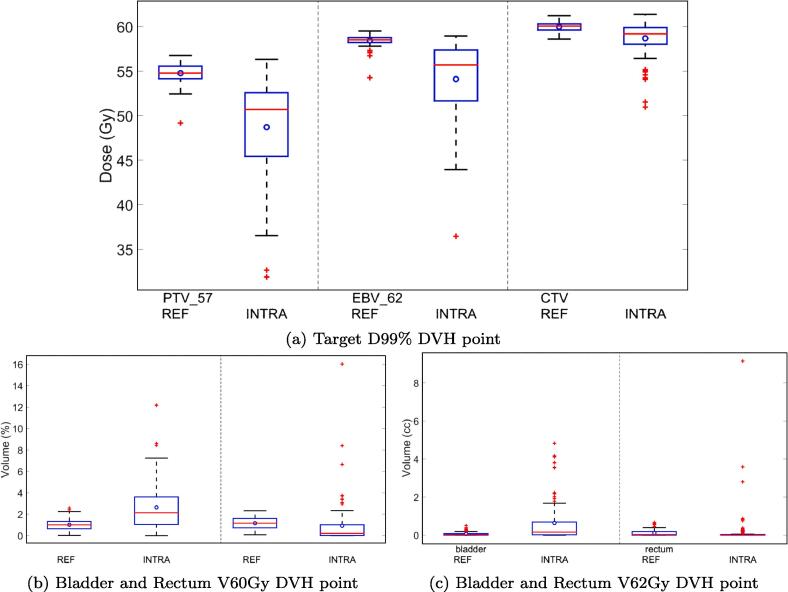
DVH boxplots for the accumulated treatment dose for each patient fraction (REF) and a total of 100 fractions (INTRA).

The average drop in D99% coverage for the PTV, EBV and CTV was 11%±9.5%, 7.4% ± 7.4% and 2.1% ± 2.9% respectively (Fig. 2a).

The V60Gy DVH point for bladder and rectum (Fig. 2b) underwent an average increase of 1.6% ± 2.3% and decrease of 0.2% ± 2.2% respectively. Similarly the V62Gy (Fig. 2c) increased for the bladder and rectum by 0.6 cm^3^ ± 1.0 cm^3^ and 0.1 cm^3^ ± 1.0 cm^3^.

### 3.4 Motion effect on dose

**Fig. 3:**
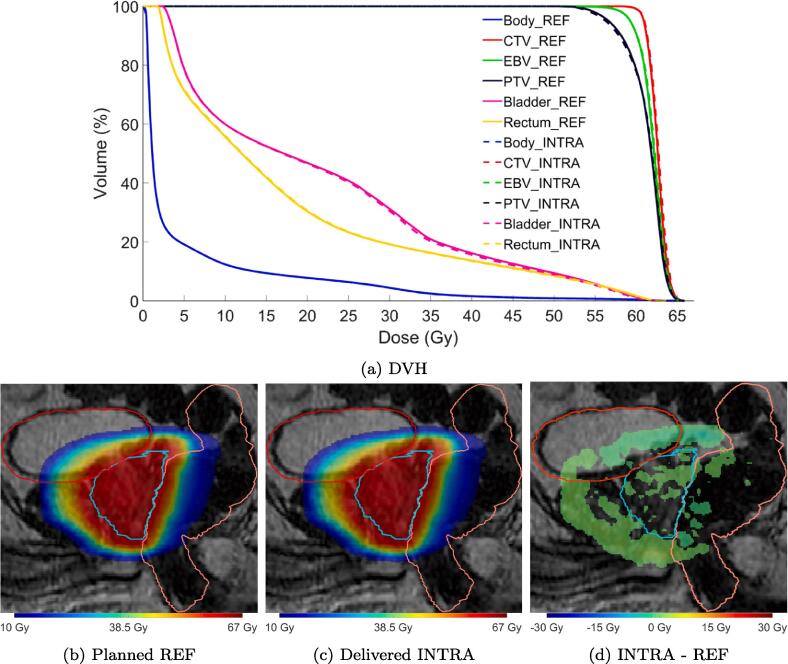
DVH (top) and sagittal slice (bottom) from the planned dose, accumulated dose and signed difference INTRA - REF for one fraction with small motion. For clearer visualization, in sub-figure c differences with absolute value less than 0.5 Gy are not shown. CTV (cyan), bladder (red) and rectum (orange) are shown.

For relatively low motion cases the dose accumulation yields an almost identical dose distribution to the original REF (Fig. 3).

**Fig. 4:**
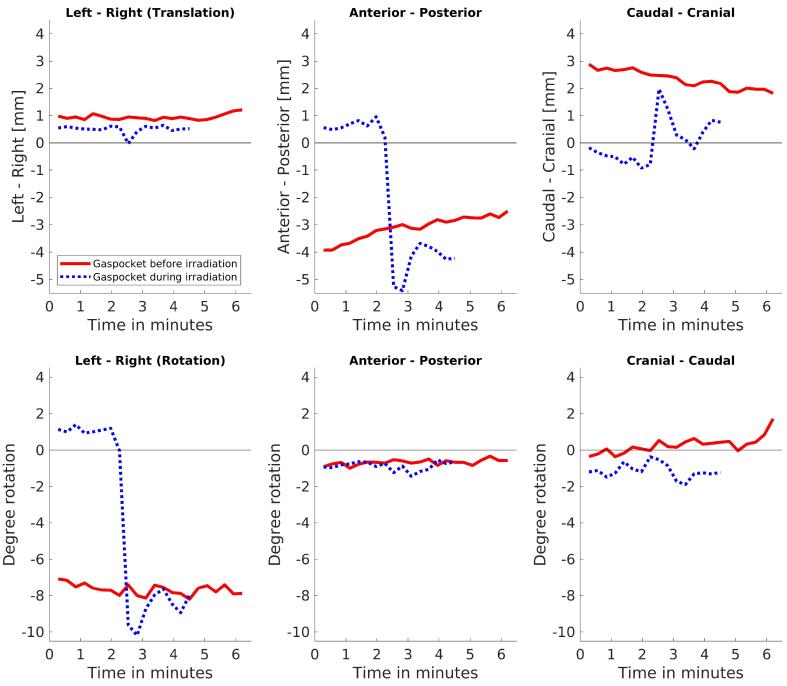
Intrafraction motion during beam-on for two fractions of individual patients with distinct traces. The graphs on the first row show the intrafraction translation, while the graphs on the second row show the intrafraction rotation. The red solid lines correspond to a case with systematic intrafraction motion while the blue dotted ones to an motion event occurring within the beam-on interval. The horizontal black lines indicate y = 0.

Depending on the magnitude, frequency and occurrence, motion can have varying effects on the clinically approved dose distributions. Fig. 4 shows the motion trace of two different patient fractions with distinct types of motion events.

**Fig. 5:**
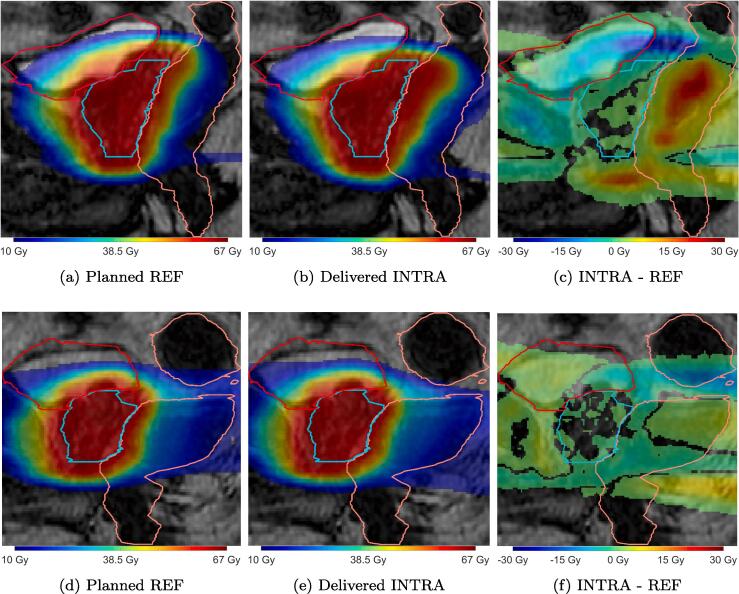
Sagittal slices from the planned dose, accumulated dose and signed difference INTRA – REF for two fractions with motion during the whole fraction (top) and mainly during beam-on (bottom). For clearer visualization, in sub-figures c and f differences with absolute value less than 0.5 Gy are not shown. CTV (cyan), bladder (red) and rectum (orange) are shown.

In a case with a systematic intrafraction shift (Fig. 4, solid), caused by a gas pocket passing through the rectum, prostate motion occurred after plan approval on the PV scan and prior to the beginning of radiation (Figs. 5c). During delivery the prostate maintained this relative offset position compared to the reference image and thus had a large impact on the dose distribution. The average rotation about the LR axis during radiation was thus −7.7 ± 0.3 degrees. The CTV D99% was reduced by 7% while rectum V60Gy was increased by 8.2%.

In another case with large motion between REF and beam-on was small, with the prostate being slightly shifted in the posterior and caudal directions during the first half of the delivery, but then —due to a rectal gas pocket—in the second half considerable motion of up to a 5.4 mm shift in the anterior direction and −10.2 degrees rotation about the LR axis was recorded (Fig. 4, dotted). Due to the interplay effect of the different beam angles and prostate positions the CTV D99% was only marginally reduced by 0.5% and rectum V60Gy decreased by 0.9% (Figs. 5f).

## 4 Discussion

In this work we present a delivered dose reconstruction method and its application on the first five prostate patients treated with online MRI-guidance in our clinic. This is the first result which combines 3D beam-on cine-MR to extract the prostate position with high frequency treatment log files to accurately reconstruct the delivered dose to the patient during each fraction.

Following the clinical treatment workflow which updates the plan based on a daily basis, we evaluated the delivered dose at a fraction by fraction basis instead of accumulating it at a single timepoint. On average the clinical margin structures were adequate to maintain the CTV dose, yielding an average drop on the D99% coverage of 2.2% ± 2.9% and a mean dose reduction of 0.3% ± 0.5%. Besides the outliers with higher impact due to rectal gas between PRE and treatment termination, a systematic shift of the prostate towards both the caudal and posterior directions of 1 mm was observed, similar to what was reported in our earlier study [17]. This was caused by increasing bladder filling during the fraction and led to the increase in bladder V62Gy by 0.6 cm^3^ ± 1 cm^3^ and decrease in rectum V62Gy by 0.1 cm^3^ ± 1 cm^3^ on average.

We utilized 3D cine-MR with high spatial and temporal frequency to extract the prostate motion with six degrees of freedom instead of 2D surrogates like implanted markers [19, ] and 2D cine-MR imaging [16, ] during each fraction. Our soft-tissue based registration method yields rigid transformations and thus limits us to prostate translation and rotation excluding the tissue deformations that take place in the surrounding organs. This is the first study to incorporate 3D-based rotations into dose reconstruction for MRI-guided treatments, which have been shown as a principal component of motion [20]. Due to the rigid information extracted exclusively from tracking the prostate region, the dynamic 3D volumes used during dose accumulation are generated by rigidly transforming the volumes of interest (VOI) while maintaining the daily body contour and bony structures in order to avoid translating/rotating the whole body anatomy [15].

In this work the dynamic cine-MR volumes were registered to the REF daily planning image acquired at the beginning of each fraction. The PV scan was only used as reference in the limited number of fractions in which a virtual couch shift was performed to correct for unacceptable intrafraction motion compared to REF. In contrast to using the PV scan as the reference of the registration [16], we incorporate the motion that occurred in the anatomy over the total time the patient was lying on the treatment couch prior to beam-on, thus yielding a better representation of the true delivered dose to the patient.

The presented results focus on the high dose region around the prostate, for both target structures and surrounding OARs. Relying on rigid motion information of the prostate, restricts the analysis of lower dose regions with increasing uncertainty as we go further away from the prostate. In order to have a more accurate and complete view of the actual delivered dose, 3D deformable registration should instead be used to track the VOI across the complete field-of-view via 3D Deformation Vector Fields (DVFs). Thorough validation of such deformable methods should be performed to ensure that the resulting DVFs do not lead to unrealistic tissue deformations [21, ] —especially when used as basis for dose accumulation, affecting the final delivered dose. We are now working towards integrating deformable registration into our dose accumulation pipeline for MRI-guided treatments.

The reported motion analysis indicates that an anisotropic margin with a reduction in the cranial direction could be feasible for this group of patients, although more patient data is needed to fully verify that before clinical application. In addition, the modification of the online protocol to always include a virtual couch shift on the PV scan prior to radiation delivery will also be investigated as it could assist a potential margin reduction. The extent of a potential margin decrease is then limited due to the outlier motion that can take always place during beam-on (Fig. 4) and demands the ability to further modify the plan during radiation delivery without too much overhead, which is not straightforward in our current clinical system.

Given the calculation speed of the various dose accumulation components and an average of 22 dynamic volumes during these patient treatments, we were able to perform image registration and dose accumulation in approximately 8.5 min for each fraction. While this timing does not include the extraction of the treatment log files and imaging data collection from the dicom storage, this method enables fast and online evaluation on a daily basis. Initially it can be used to assess the delivered dose after treatment completion and eventually to monitor the delivered dose during beam-on. We are working towards integrating both registration and dose components into a unified application that will allow for a fully automated delivered dose pipeline.

After analysing the first prostate MR-linac patients in our clinic, we would like to apply the dose reconstruction method on the hypofractionated MRI-guided treatments currently performed, which involve five fractions of 7.25 Gy instead of 20x3.1 Gy. In this setting tracking the anatomical motion in 3D and coupling it to our dose accumulation pipeline becomes more critical to establish the accurate dose delivery due to the longer beam-on time and fewer fractions.

In this study we presented the first delivered dose quantification during MRI-guided prostate radiotherapy based on 3D cine-MR with high temporal frequency. We developed a fast and easy to automate dose reconstruction pipeline combining six degrees of freedom motion and radiotherapy treatment log files to accurately reconstruct the dose delivered during every treatment fraction. These results demonstrate that the current clinical margins for these 20x3.1 Gy prostate patients are adequate and lead to the intended delivered dose, although specific outlier fractions with large motion due to rectal/bladder filling can experience significant dose deviations. An accurate dose reconstruction method is essential as we progress to more hypofractionated treatments in order to evaluate the true delivered dose distribution and integrate it into adaptive real-time planning methods able to modify the plan during radiation delivery.

## Declaration of Competing Interest

The authors declare that they have no known competing financial interests or personal relationships that could have appeared to influence the work reported in this paper.
