# Supplementary material for: Delivered dose quantification in prostate radiotherapy using online 3D cine imaging and treatment log files on a combined 1.5T magnetic resonance imaging and linear accelerator system
Source: Phys Imaging Radiat Oncol. 2020 Jul 13;15:23–9. doi: 10.1016/j.phro.2020.06.005 (PMC7807644; doi:10.1016/j.phro.2020.06.005)
Supplement: Supplementary data [file mmc1.pdf]

Supplementary material

Table S1: Imaging parameters used for the acquisition of the cine-MR images for the first 88 fractions. The images were acquired with a 3D balanced turbo field echo (bTFE) with fat suppression.

**Description**	**Value**
Sequence	bTFE with fat suppression
Time per dynamic	16.9 seconds
Relaxation time T_R	4.7 ms
Echo time T_E	2.3 ms
Field strength B_0	1.5T
Flipangle	50°
Field of view	448 x 448 x 63 voxels
Acquired voxel spacing	2 x 2 x 2 mm
Reconstructed voxel spacing	0.9 x 0.9 x 2 mm
Bandwidth	434 Hz/px

Table S2: Imaging parameters used for the improved acquisition of the cine-MR images for the last 12 fractions. The images were acquired with a 3D balanced turbo field echo (bTFE) without fat suppression.

**Description**	**Value**
Sequence	bTFE without fat suppression
Time per dynamic	8.6 seconds
Relaxation time T_R	4.7 ms
Echo time T_E	2.3 ms
Field strength B_0	1.5T
Flipangle	50°
Field of view	512 x 512 x 45 voxels
Acquired voxel spacing	2 x 2 x 2.2 mm
Reconstructed voxel spacing	0.8 x 0.8 x 2.2 mm
Bandwidth	434 Hz/px

Table S3: Imaging parameters used for the T2-weighed pre-treatment (PRE), position verification (PV) and post-treatment (Post) scans.

**Description**	**Value**
Sequence	T2-weighted 3D
Acquisition duration	116.7 seconds
Repetition time	1535 ms
Echo time T_E	277.8 ms
Field strength B_0	1.5T
Flipangle	90°
Acquisition field of view	400 x 400 x 300 voxels
Reconstructed field of view	480 x 480 x 480 voxels
Acquired voxel spacing	1.5 x 1.5 x 2 mm
Reconstructed voxel spacing	0.83 x 0.83 x 1.0 mm
Bandwidth	740 Hz/px
Slice spacing	1 mm
SENSE factor	3.6 (RL)
Partial Fourier (Halfscan Y)	0.62
